# Polypharmacy and Drug–Drug Interaction Architecture in Hospitalized Cardiovascular Patients: Insights from Real-World Analysis

**DOI:** 10.3390/biomedicines14010218

**Published:** 2026-01-20

**Authors:** Andrei-Flavius Radu, Ada Radu, Gabriela S. Bungau, Delia Mirela Tit, Cosmin Mihai Vesa, Tunde Jurca, Diana Uivarosan, Daniela Gitea, Roxana Brata, Cristiana Bustea

**Affiliations:** 1 Doctoral School of Biological and Biomedical Sciences, University of Oradea, 410087 Oradea, Romania; andreiflavius.radu@uoradea.ro (A.-F.R.); dtit@uoradea.ro (D.M.T.); cosmin.vesa@csud.uoradea.ro (C.M.V.); tjurca@uoradea.ro (T.J.); cbustea@uoradea.ro (C.B.); 2 Department of Psycho-Neuroscience and Recovery, Faculty of Medicine and Pharmacy, University of Oradea, 410073 Oradea, Romania; 3 Department of Pharmacy, Faculty of Medicine and Pharmacy, University of Oradea, 410028 Oradea, Romania; dgitea@uoradea.ro; 4 Department of Preclinical Disciplines, Faculty of Medicine and Pharmacy, University of Oradea, 410073 Oradea, Romania; diana.uivarosan@didactic.uoradea.ro; 5 Department of Medical Disciplines, Faculty of Medicine and Pharmacy, University of Oradea, 410073 Oradea, Romania; roxana.gavrila@yahoo.com

**Keywords:** drug–drug interactions, cardiovascular disease, drug interaction checker, polypharmacy, real-world data, network analysis, drug safety

## Abstract

**Background:** Cardiovascular polypharmacy inherently amplifies the risk of drug–drug interactions (DDIs), yet most studies remain limited to isolated drug pairs or predefined high-risk classes, without mapping the systemic architecture through which interactions accumulate. **Objectives:** To characterize the burden, severity, and network structure of potential DDIs in a real-world cohort of hospitalized cardiovascular patients using interaction profiling combined with graph-theoretic network analysis. **Methods:** This retrospective observational study included 250 hospitalized cardiovascular patients. All home medications at admission were analyzed using the Drugs.com interaction database, and a drug interaction network was constructed to compute topological metrics (i.e., degree, betweenness, and eigenvector centrality). **Results:** Polypharmacy was highly prevalent, with a mean of 7.7 drugs per patient, and 98.4% of patients exhibited at least one potential DDI. A total of 4353 interactions were identified, of which 12.1% were classified as major, and 35.2% of patients presented high-risk profiles with ≥3 major interactions. Interaction burden showed a strong correlation with medication count (r = 0.929). Network analysis revealed a limited cluster of hub medications, particularly pantoprazole, furosemide, spironolactone, amiodarone, and perindopril, that disproportionately governed both interaction density and high-severity risk. **Conclusions:** These findings move beyond conventional pairwise screening by demonstrating how interaction risk propagates through interconnected therapeutic networks. The study supports the integration of hub-focused deprescribing, targeted monitoring strategies, and network-informed clinical decision support to mitigate DDI risk in cardiovascular polypharmacy. Future studies should link potential DDIs to clinical outcomes and validate network-based prediction models in prospective settings.

## 1. Introduction

Cardiovascular diseases (CVDs) remain the foremost contributor to mortality worldwide, accounting for a substantial proportion of global health loss. In 2022, these conditions were responsible for an estimated 19.8 million deaths, which corresponds to nearly one-third of all deaths worldwide [1]. Among the CVDs most frequently encountered and associated with substantial morbidity and mortality are ischemic heart disease, hypertension, stroke and other cerebrovascular syndromes, heart failure, atrial fibrillation, pulmonary and systemic thromboembolic diseases, and peripheral arterial disease [2]. 

Although age-adjusted cardiovascular rates are expected to stabilize or decline, the absolute number of CVD events is projected to increase substantially between 2025 and 2050 due to global population aging [3]. Population aging and multimorbidity expand guideline-directed regimens, and the growing uptake of pleiotropic cardiometabolic agents (i.e., SGLT2 inhibitors and GLP-1 receptor agonists) further increases medication counts and the probability of interaction-related harm [4]. 

Current CVD management relies on complex, guideline-directed combination therapy, with most patients receiving multiple cardiovascular and non-cardiovascular medications simultaneously. Standard regimens typically include antiplatelet agents (acetylsalicylic acid), high-intensity statins (i.e., atorvastatin), renin–angiotensin system blockers (i.e., perindopril), and beta blockers (i.e., bisoprolol). Patients with heart failure additionally receive loop diuretics (i.e., furosemide) and mineralocorticoid receptor antagonists (i.e., spironolactone). Many also require oral anticoagulants for atrial fibrillation and proton pump inhibitors (i.e., pantoprazole) for gastroprotection alongside therapies. Consequently, guideline-based cardiovascular care systematically generates multimodal treatment schemes in which polypharmacy is inevitable and the potential for clinically relevant drug–drug interactions (DDIs) is structurally embedded [5,6,7,8]. 

Polypharmacy is a fundamental driver of DDI risk in cardiovascular care, as patients with CVD frequently accumulate multiple therapies. The absence of a universal definition and the wide variation in thresholds complicate its clinical interpretation, yet most older adults with CVD routinely exceed five concurrent medications. Multimorbidity, prescribing cascades, fragmented care, and limited trial data in elderly and female populations further amplify medication load [9]. As the number of concomitant medications increases, the chance of potential DDIs escalates sharply, increasing from an estimated 6% with two concomitant medications to approximately 50% with five agents and becoming almost 100% when eight drugs are co-administered [10]. 

Individuals with CVDs, who routinely require such extensive treatment regimens, consequently exhibit a markedly heightened susceptibility to interaction-related harm and hospitalization [11]. Clinically important DDIs are frequent in cardiovascular care, where multiple high-risk combinations can compromise safety. Interactions that intensify central nervous system depression, prolong the QT interval, or alter gastrointestinal drug absorption commonly complicate therapy. Particularly hazardous pairs include non-selective beta-blockers that counteract β2-agonist bronchodilation and anticholinergic agents that heighten the ulcerogenic potential of solid oral potassium [12]. Cardiovascular-specific risks also arise from additive bradycardic effects (i.e., beta-blockers with digoxin) and from agents that disturb electrolyte balance, creating conditions that can precipitate arrhythmias or conduction block [13]. 

Mitigating interaction risks requires systematic regimen reviews, distinguishing appropriate polypharmacy, structured deprescribing, vigilant clinical monitoring, and multidisciplinary coordination to align therapy with patient-specific goals [14,15]. Yet systematic understanding of interaction accumulation in polypharmacy remains lacking, requiring network-level analysis [15]. 

However, despite the widespread availability of DDI screening tools and established principles for risk mitigation, existing approaches remain largely pairwise and fail to capture how interactions systematically accumulate, overlap, and propagate within complex multidrug regimens [16,17]. This leaves a critical gap in understanding the emergent properties of polypharmacy: specifically, which medications disproportionately drive overall interaction burden, how risks cluster around certain drug combinations, and which agents contribute most to high-severity interaction profiles in real-world cardiovascular practice. Without this systems-level perspective, clinicians lack the evidence needed to prioritize deprescribing efforts or identify the most problematic contributors in patients receiving multiple interacting medications simultaneously.

Digital online tools designed to detect DDIs by identifying individual drug pairs, classifying interaction severity, and providing standardized management recommendations at the point of care [12,15] have become essential in cardiovascular pharmacotherapy, where complex regimens amplify interaction risk. These interaction checkers provide structured, continuously updated assessments that support clinicians in identifying hazardous combinations and guide them in refining therapeutic decisions. Tools capable of generating full interaction profiles for single agents, notably Drugs.com, are particularly valuable in polypharmacy-heavy cardiovascular care. When integrated into clinical workflows, these resources enhance medication safety by flagging high-severity interactions, guiding monitoring needs, and aiding the optimization of multidrug regimens [18]. 

The present study aims to characterize the burden, severity, and structural organization of potential DDIs in a real-world cohort of hospitalized cardiovascular patients with polypharmacy. By integrating comprehensive interaction profiling with graph-theoretic network analysis, the study moves beyond isolated drug-pair assessments to identify key medications and interaction clusters that disproportionately shape overall interaction burden and high-severity risk. This approach provides a foundation for improved risk stratification, therapeutic optimization, and a more precise acknowledgment of the clinical relevance of DDIs in complex cardiovascular pharmacotherapy.

The manuscript is structured as follows: Section 2 describes the study design, data collection, and the interaction and network analysis workflow, followed in Section 3 by the presentation of the descriptive and network-based results; Section 4 discusses these findings in the context of current evidence and clinical implications, and Section 5 concludes by summarizing the main conclusions and future directions.

## 2. Materials and Methods

### 2.1. Study Design and Data Collection

The present research was conducted as a retrospective observational analysis of DDIs in adult patients hospitalized for cardiovascular diseases. The study included 250 adult patients admitted for cardiovascular conditions, including coronary artery disease, heart failure, arrhythmias, prior myocardial infarction, peripheral arterial disease, venous thrombosis, atrial fibrillation, and hypertension, at the County Emergency Clinical Hospital Oradea, Romania.

Demographic data (i.e., age, sex, and residence), clinical diagnoses, and complete medication lists at hospital admission were extracted retrospectively from electronic medical records. Data were collected for all eligible patients admitted between June 2024 and January 2025.

As this was a descriptive observational study focused on characterizing DDIs burden, severity patterns, and network topology, rather than testing specific hypotheses or comparing groups, no formal a priori sample size calculation was performed. The sample size was determined by consecutive enrollment of all eligible patients admitted during the predefined study period (June 2024–January 2025). The resulting cohort of 250 patients provided narrow confidence intervals for key prevalence estimates (e.g., 95% CI for overall DDI prevalence: 95.9–99.5%, Clopper-Pearson exact method) and yielded a sufficiently diverse medication pool (110 unique drugs generating 4353 interactions) for robust network analysis and centrality metric estimation.

Inclusion criteria were: (i) adult patients (≥18 years); (ii) hospitalization for a primary cardiovascular diagnosis; (iii) availability of a complete medication list at hospital admission; and (iv) prescription of at least two pharmacologically active agents at admission. 

Exclusion criteria were limited to: (i) incomplete or missing medication records and (ii) inability to identify active substances for prescribed drugs. No additional exclusion criteria were applied to preserve real-world representativeness of cardiovascular polypharmacy.

Study endpoints were predefined and descriptive in nature. The primary endpoint was the total burden of potential DDIs per patient at admission. Secondary endpoints included: (i) distribution of DDI severity categories (minor, moderate, major); (ii) identification of medications disproportionately contributing to overall and major-severity DDI burden; (iii) characterization of recurrent interacting drug pairs; and (iv) structural properties of the DDI network, including centrality metrics, clustering, and network density.

### 2.2. Medication Classification and DDI Assessment

DDIs were identified using the Drugs.com interaction database [19] accessed on 18 December 2025, a widely used and regularly updated clinical decision-support resource that integrates evidence from regulatory sources, primary literature, and expert curation. The database provides standardized interaction severity classification (i.e., minor, moderate, major), together with mechanistic explanations and management recommendations, and has demonstrated high sensitivity for the detection of clinically significant DDIs in comparative evaluations. All drug–drug interaction outputs generated from the database were independently screened and validated by two authors (A.-F.R. and C.B.). Any discrepancies in severity assignment or pair identification were resolved through joint review to ensure consistency and minimize classification error. Interactions were classified according to the platform’s severity categories. These interaction categories reflect escalating levels of clinical relevance, ranging from minor interactions, which carry minimal therapeutic consequence, to moderate interactions, which may necessitate closer monitoring or dose adjustments to maintain safety and efficacy, and up to major interactions, which are potentially clinically significant or contraindicated and may require prompt modification or complete avoidance of the drug combination.

For each patient, total interaction count, number of major interactions, and proportions of interaction categories were computed. To ensure full concordance between individual-level metrics and aggregate severity distributions, all interaction counts were computed directly from the standardized drug lists for each patient.

### 2.3. Network Analysis

A comprehensive drug interaction network was constructed and analyzed using NetworkX [20] version 3.5, an open-source Python library designed for the creation, analysis, and visualization of complex networks, to characterize structural patterns and identify hub medications within the cohort’s polypharmacy landscape. NetworkX provides validated implementations of graph-theoretic algorithms, including centrality measures, clustering metrics, and component analysis, and is widely used in biomedical, pharmacological, and systems medicine research.

Nodes represented individual medications, and undirected edges indicated documented interactions between drug pairs. Edge weights were assigned based on interaction severity using a predefined numerical mapping: Major = 3, Moderate = 2, Minor = 1, Unknown = 0.5. When multiple interactions existed between the same drug pair across different patients, weights were summed and interaction counts aggregated. 

Network centrality metrics were computed using established NetworkX algorithms to identify structurally important medications. Degree centrality was calculated as the number of direct connections per drug node. Betweenness centrality was computed using nx.betweenness_centrality [21] to quantify how frequently each drug appeared on shortest paths between other drug pairs, indicating bridging roles in the network. Closeness centrality was calculated using nx.closeness_centrality [22] to measure the inverse of average shortest path distance to all other drugs, reflecting diffusion potential. Eigenvector centrality was computed using nx.eigenvector_centrality [23] (max_iter = 1000) to assess node influence based on connections to other well-connected drugs. 

Network topology was characterized through clustering analysis and component detection. Local clustering coefficients were computed using nx.clustering [24] to quantify the proportion of each drug’s neighbors that were also connected to each other. The global average clustering coefficient was calculated using nx.average_clustering [25]. Connected components were identified using nx.connected_components [26], and overall network density was computed using nx.density [27] to assess the proportion of realized connections relative to all possible edges.

Network metrics were visualized across four panels: degree distribution (histogram, 50 bins), top 20 hub drugs ranked by degree (horizontal bar chart), top 15 drugs by betweenness centrality (horizontal bar chart), and clustering coefficient distribution (histogram, 30 bins). 

Network topology was further visualized by constructing a subgraph containing the 25 drugs with highest degree centrality and all edges connecting them. Node positions were determined using the Kamada-Kawai force-directed layout algorithm (nx.kamada_kawai_layout) [28], which minimizes edge crossing and positions highly connected nodes centrally through iterative energy minimization. Node size was scaled proportionally to degree centrality (size = degree × 100), and node color was mapped to betweenness centrality using a continuous yellow-orange-red color gradient (matplotlib colormap ‘YlOrRd’), with darker colors indicating higher betweenness values. Edge width was scaled proportionally to the number of patient-level interactions observed between each drug pair (width = interaction count × 0.5). Drug labels were positioned at node centers with semi-transparent white background boxes to ensure readability. The visualization included a color bar legend for betweenness centrality interpretation and a size reference legend showing node degree scaling. Network density and average clustering coefficient were computed for the subgraph and displayed as annotated statistics.

Network analysis metrics and their implementation are summarized in Table 1.

### 2.4. Statistical Analysis

Demographic characteristics were analyzed using Python 3.12.3 with pandas (≥1.3.0), numpy (≥1.21.0), matplotlib (≥3.3.0), and seaborn (≥0.11.0). Age distribution was visualized as a histogram with 20 bins, and summarized using mean, standard deviation, median, and range. Sex distribution was expressed as counts and percentages and displayed as a pie chart. All figures were configured using matplotlib with seaborn-v0_8 styling and the following parameters: 300 DPI resolution, 12 pt default font size, 14 pt subplot titles, 12 pt axis labels, and 10 pt tick labels. 

Medication burden per patient was calculated as the total number of prescribed medications at admission. The distribution was visualized as a histogram with 20 bins and summarized using mean, median, and range. Patients were further categorized into three medication burden groups using pandas.cut: low burden (1–5 medications), medium burden (6–10 medications), and high burden (≥11 medications). The proportion of patients in each category was displayed as a pie chart with percentage labels. 

Drug interaction frequencies were calculated by extracting all drug names from both positions (Drug1 and Drug2) in the detailed interactions dataset. Frequency counts for each unique medication were computed using Python’s collections. Counter and organized in descending order. The top 20 medications by interaction count were visualized as a horizontal bar chart with value labels. The overall distribution of interaction frequencies across all 110 unique medications was displayed as a histogram with 50 bins. Summary statistics (mean, median, total unique drugs, and most interactive medication) were computed using pandas methods and displayed as an annotated text box. 

The relationship between medication count and total interaction burden was visualized as a scatter plot (alpha = 0.6, marker size = 50) with a linear regression trend line fitted using numpy.polyfit (degree = 1). The Pearson correlation coefficient was computed using pandas.DataFrame.corr and displayed as an annotated text box. Patients were categorized into interaction burden groups: low (0–10 interactions), medium (11–30 interactions), and high (≥31 interactions). The distribution across these categories was displayed as a bar chart.

Overall severity distribution was computed by aggregating all identified interactions and categorizing them according to their assigned severity level (Minor, Moderate, Major). Severity frequencies were calculated using pandas.Series.value_counts. The distribution was visualized as both a pie chart (with percentage labels, start angle = 90°) and a bar chart (with absolute count labels positioned above each bar). Severity distribution patterns were analyzed for the 15 most frequently interacting medications. All interactions involving each of these drugs (either as Drug1 or Drug2) were identified and their severity levels recorded. For each medication, interactions were grouped by severity category using pandas.DataFrame.groupby with pandas.Series.unstack (fill_value = 0) to create a cross-tabulation matrix. Percentage distributions were calculated by dividing absolute counts by row sums. Both absolute counts and percentages were visualized as stacked vertical bar charts (pandas.DataFrame.plot with kind = ‘bar’, stacked = True). Patient-level risk stratification was performed based on the number of major interactions per patient. Three risk categories were defined: low risk (0 major interactions), moderate risk (1–2 major interactions), and high risk (≥3 major interactions). Risk category distribution was visualized as a pie chart with percentage labels. The distribution of major interactions per patient was displayed as a histogram (15 bins) with summary statistics (mean, median, maximum) in an annotated text box. The relationship between total interaction count and major interaction count was examined using a scatter plot (alpha = 0.6, marker size = 50), with Pearson correlation coefficient computed using pandas.DataFrame.corr and displayed as an annotation. The distribution of major interaction percentage (major interactions as proportion of total interactions per patient) was visualized as a histogram (15 bins). 

This study employed a descriptive analytical framework focused on characterizing DDI burden, severity distribution, and network topology in a real-world cohort. Given that the primary aim was comprehensive enumeration and structural characterization rather than hypothesis testing about group differences or intervention effects, no inferential statistical tests (e.g., *t*-tests, ANOVA, chi-square tests) were applied, and no *p*-values were computed. Network metrics were derived using graph-theoretic algorithms as described in Section 2.3.

### 2.5. Ethical Approval

The study was conducted in full compliance with the ethical standards of the Declaration of Helsinki and was authorized by the Ethics Committee of the Bihor County Emergency Clinical Hospital in Romania, which granted approval under number 38081 on 11 December 2025.

## 3. Results

The cohort of 250 patients was stratified by sex to assess potential disparities in clinical presentation. As shown in Table 2, the population was composed of 139 males (55.6%) and 111 females (44.4%). Rural residence was more frequent overall, accounting for 60.8% of the cohort.

A statistically significant difference in age was observed between groups (*p* = 0.002); female patients were older on average (71.08 ± 11.85 years) compared to males (66.03 ± 13.13 years). Despite the age difference, the medication burden was comparable between sexes (*p* = 0.30), with males prescribed a mean of 7.86 ± 2.85 medications and females 7.50 ± 2.58 medications.

Heart failure was the most frequent admission diagnosis (22.8%), followed by unstable angina (20.8%) and pulmonary embolism (13.2%). While the overall distribution of diagnoses did not differ significantly by sex (*p* = 0.24), distinct clinical patterns were noted: pulmonary embolism was notably more frequent in females (15.3%) compared to males (7.2%), whereas acute myocardial infarction was more common in the male cohort (13.7% vs. 9.9%).

**Figure 1:**
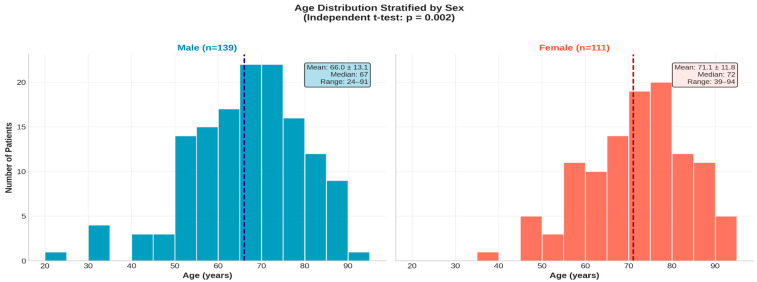
Age distribution of the study cohort stratified by sex (*n* = 250). (**Left panel**): age distribution in male patients (*n* = 139); (**Right panel**): age distribution in female patients (*n* = 111). The dashed vertical line in each histogram represents the median age of that group 67 years for males and 72 years for females.

The age profile of the 250 patients demonstrated a broad distribution ranging from 24 to 94 years, with a notable concentration of individuals in the seventh and eighth decades of life. Stratification by sex revealed distinct demographic patterns: while male patients represented 55.6% of the cohort, they exhibited a wider age distribution extending into younger decades (mean 66.0 ± 13.1 years). In contrast, the female population (44.4%) was significantly older and predominantly clustered in the advanced age groups, with a mean age of 71.1 ± 11.8 years. These divergent age structures are illustrated in the stratified histograms in Figure 1.

**Figure 2:**
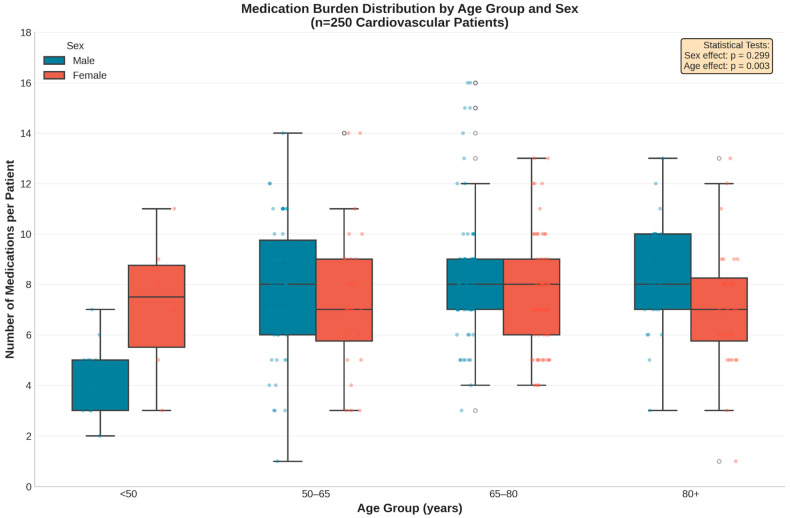
Distribution of medication burden stratified by age group and sex. The box plots reveal a significant age-dependent increase in medication count among male patients (*p* = 0.003), whereas female patients exhibited a consistently high medication burden across all age categories. Blue = Male; Red = Female. Overlaid points represent individual patient-level observations (jittered for visibility). Center line = median; Box limits = interquartile range; Whiskers = range (excluding outliers).

Medication burden varied significantly by age (*p* = 0.003) with a notable age × sex interaction (*p* = 0.002; Figure 2). Male patients exhibited a clear age-dependent increase in polypharmacy, whereas female patients maintained consistently high medication counts across all age groups. This pattern suggests that factors driving polypharmacy accumulation differ between sexes. Dispersion of medication counts increased in older age groups, as reflected by wider interquartile ranges and higher maximum values, particularly among males.

**Figure 3:**
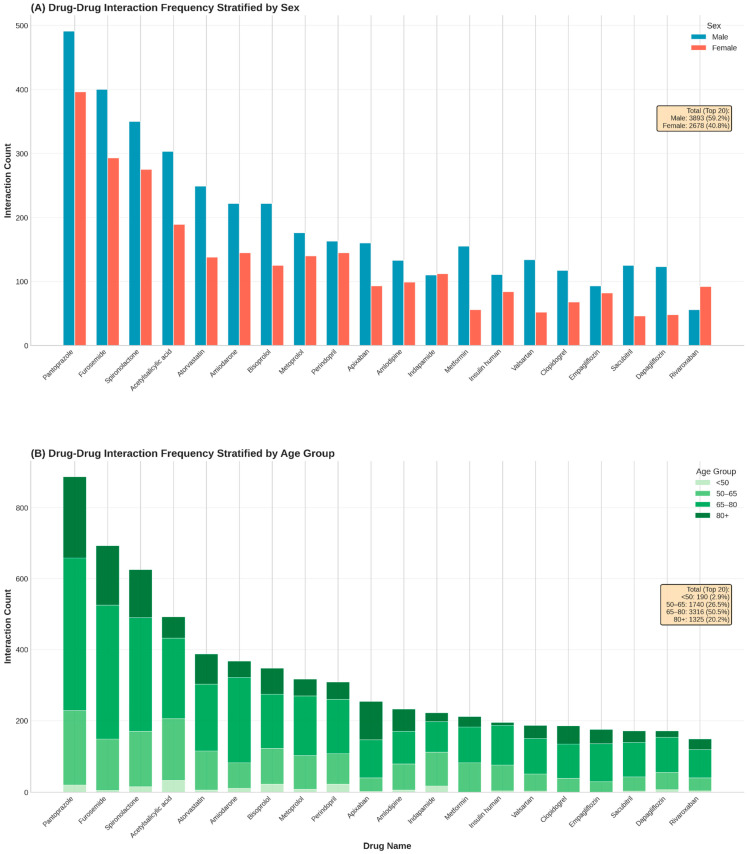
Stratification of the top 20 medications involved in drug–drug interactions by patient demographics. (**A**): Absolute frequency of interactions for the top 20 drugs stratified by sex. (**B**): Proportional distribution of interaction burden across four age categories.

Analysis of the top 20 medications contributing to potential DDIs revealed distinct demographic footprints defined by patient sex and age (Figure 3). Pantoprazole remained the leading source of interactions overall (*n* = 887), followed by furosemide (*n* = 693) and spironolactone (*n* = 625).

Stratification by sex (Figure 3, top panel) generally reflected the baseline male predominance in the cohort, with men accounting for the majority of interactions for most top-ranked drugs. High male-to-female ratios were particularly evident for metabolic and heart failure agents, including metformin (2.77), sacubitril (2.72), and dapagliflozin (2.56). However, notable exceptions indicated sex-specific prescribing patterns: interactions involving the diuretic indapamide were evenly distributed (male/female ratio 0.98), while the anticoagulant rivaroxaban exhibited a distinct female predominance (*n* = 92 vs. *n* = 56; ratio 0.61), diverging from the trend observed with apixaban (*n* = 160 vs. *n* = 93; ratio 1.72).

Stratification by age (Figure 3, bottom panel) demonstrated pharmacological shifts associated with aging. The interaction burden for core heart failure and diuretic therapies was heavily concentrated in older cohorts; for furosemide, pantoprazole, and spironolactone, over 70% of all interactions occurred in patients aged ≥65 years. Specifically, apixaban interactions were disproportionately frequent in the oldest-old cohort (>80 years), which accounted for 42.3% of its total burden. In contrast, interactions involving indapamide were concentrated in the middle-aged group (50–65 years; 42.8%), reflecting the distinct therapeutic focus of hypertension management in younger seniors compared to the complex multimorbidity regimens of the very elderly.

Analysis of drug pair frequencies identified 911 unique combinations with a highly right-skewed distribution. Atorvastatin + pantoprazole, furosemide + pantoprazole, and pantoprazole + spironolactone were the most frequent pairs, reflecting the central role of gastroprotection in polypharmacy regimens.

**Figure 4:**
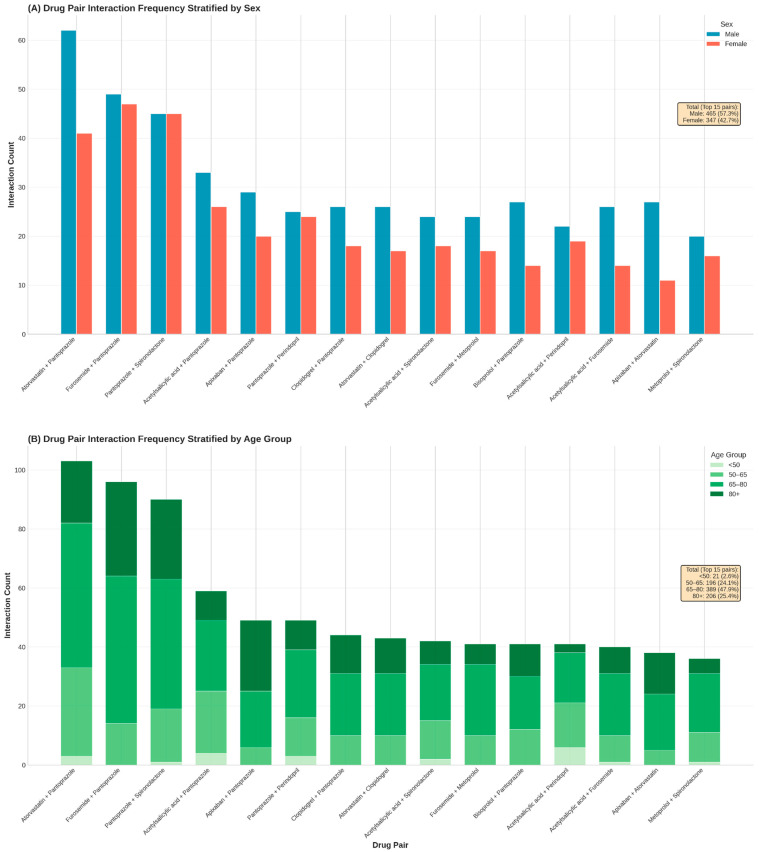
Stratification of the most frequent drug–drug interaction pairs by patient demographics. (**A**): Frequency of the top 15 recurrent drug pairs stratified by sex. (**B**): Proportional distribution of these high-frequency pairs across four age categories (<50, 50–64, 65–79, and ≥80 years), highlighting age-specific accumulation of specific therapeutic combinations.

Figure 4 illustrates the distribution of the most frequent interacting drug pairs stratified by sex (Panel A) and age group (Panel B). Overall, male patients accounted for a higher proportion of interaction pairs than females among the top-ranked combinations, particularly for statin–PPI and diuretic–PPI pairs. Age-stratified analysis showed a progressive increase in interaction frequency with advancing age, with the highest counts observed in patients aged ≥ 65 years. The <50-year group contributed minimally to the overall interaction burden, whereas patients aged 65–80 and ≥80 years accounted for the majority of recurrent drug pairs.

The relationship between the number of prescribed medications and the corresponding interaction burden was characterized by a strong positive linear association. Across the patient cohort, the number of medications ranged from 1 to 16, and the total number of identified interactions per patient increased proportionally with medication count. The scatterplot exhibited a dense ascending pattern of points along the fitted regression line, with a correlation coefficient of r = 0.929, indicating a close alignment between the number of medications and interaction frequency. This correlation coefficient was computed automatically in Python using standard numerical libraries, without the application of hypothesis testing or inferential statistical procedures. Individual interaction counts extended to nearly 80 interactions in patients with the highest medication numbers.

**Figure 5:**
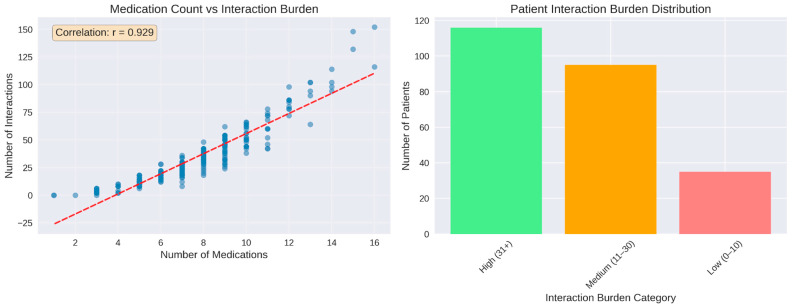
Medication count as predictor of interaction burden. (**Left panel**): strong positive correlation be-tween number of medications and total interactions (r = 0.929). (**Right panel**): distribution of patients across interaction burden. The red dashed line represents a linear regression trend line that illustrates the relationship between the key variables.

Classification of patients according to interaction burden revealed that the medium-burden group (11–30 interactions) represented the largest category, comprising approximately 126 patients. The low-burden group (0–10 interactions) included around 88 patients, while the high-burden category (31 or more interactions) consisted of 34 patients (Figure 5). In addition to medication count, patient age was associated with higher interaction burden, with older age groups exhibiting increased numbers of potential DDIs, as shown in age-stratified analyses. Sex-related differences were observed in absolute interaction counts, largely reflecting differences in medication exposure rather than intrinsic sex-specific risk. No additional systematic associations were evaluated for other patient characteristics such as smoking status, alcohol consumption, allergies, or comorbidities, as these variables were not consistently documented or were infrequent in the cohort. Consequently, interaction burden in this analysis was primarily driven by medication number and age-related prescribing complexity.

**Figure 6:**
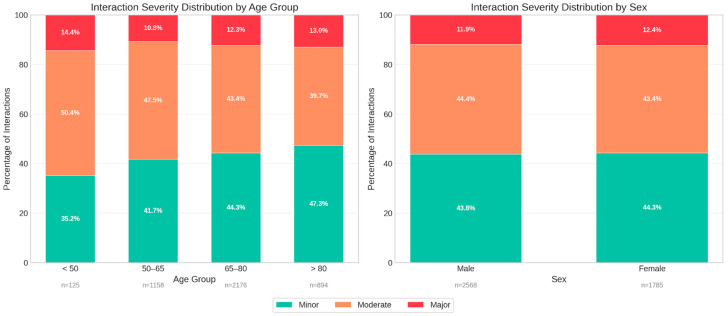
Stratification of drug–drug interaction severity profiles by patient demographics. (**Left panel**): Proportional distribution of interaction severity (minor, moderate, major) across four age groups (<50, 50–65, 65–80, and >80 years), (**Right panel**): Severity distribution stratified by sex, demonstrating comparable risk profiles between male and female patients.

Stratification of interaction severity by patient characteristics revealed distinct age-related patterns (Figure 6). Contrary to the assumption that advanced age drives interaction severity, the youngest cohort (<50 years) exhibited the highest relative proportion of major interactions (14.4%), followed by the >80 age group (13.0%). The <50 cohort also displayed the highest proportion of moderate interactions (50.4%), whereas patients >80 years showed a shift toward minor interactions (47.3%).

In terms of absolute volume, the 65–80 age group accounted for the largest number of total interactions (*n* = 2176). Sex-based analysis indicated no clinically significant differences in severity profiles, with males and females demonstrating nearly identical distributions of major (11.9% vs. 12.4%), moderate (44.4% vs. 43.4%), and minor (43.8% vs. 44.3%) interactions. This suggests that while age significantly influences the composition of interaction risk, sex is not a primary determinant of interaction severity within this cardiovascular setting.

Additional patient-level severity metrics provided further granularity beyond the aggregate distribution. A total of 183 patients (73.2%) experienced at least one major interaction, while moderate and minor interactions occurred in 238 (95.2%) and 235 patients (94.0%), respectively. Furthermore, 88 individuals (35.2%) met the criterion for high-risk exposure, defined as having three or more major interactions. The mean number of major interactions per patient was 2.1, with a median of 2 and a maximum of 13 observed in a single case. Minor and moderate interactions occurred at comparable average levels (7.7 per patient for each category). In contrast, 67 patients (26.8%) had no major interactions, reflecting the heterogeneous severity distribution across the cohort.

Among the 15 medications with the highest interaction counts, the distribution of severity levels demonstrated substantial quantitative variation. In absolute terms, acetylsalicylic acid registered a total of approximately 480 interactions, composed of large contributions from moderate events, followed by minor and a smaller proportion of major interactions. Amiodarone accounted for roughly 330 interactions, with moderate interactions representing the predominant category, alongside a substantial minor component and a smaller number of major events. Amlodipine showed approximately 215 interactions, distributed mainly between moderate and minor categories, with major interactions forming a minimal share. Apixaban exhibited around 220 interactions, with moderate interactions constituting the majority, minor interactions forming the secondary group, and major interactions appearing infrequently. Atorvastatin recorded nearly 290 interactions with a pattern dominated by moderate events, accompanied by minor interactions and a comparatively small number of major interactions. Bisoprolol presented approximately 320 interactions, primarily moderate and minor, with a very small fraction classified as major. Furosemide had more than 550 interactions in total, the highest within the group, with large contributions from both moderate and minor categories and a smaller number of major interactions. Indapamide showed approximately 170 interactions, predominantly moderate, followed by minor interactions, and only a small number of major events. Insulin human and metformin showed lower absolute counts relative to other medications in the group, with both drugs presenting mainly moderate interactions and very few major events. Metoprolol exhibited around 230 interactions, composed predominantly of moderate and minor interactions, with a smaller number of major events. Pantoprazole had more than 330 interactions, with minor interactions forming the largest share, followed by moderate interactions and a small proportion of major interactions. Perindopril demonstrated approximately 390 interactions, with minor interactions representing the dominant category, followed by moderate interactions and a considerable number of major interactions relative to the rest of the group. Spironolactone recorded more than 350 interactions, again showing a strong predominance of minor interactions, followed by moderate interactions and a notable count of major interactions. Valsartan showed a lower overall number of interactions, with the distribution consisting mainly of moderate events, followed by minor interactions and a smaller number of major interactions.

**Figure 7:**
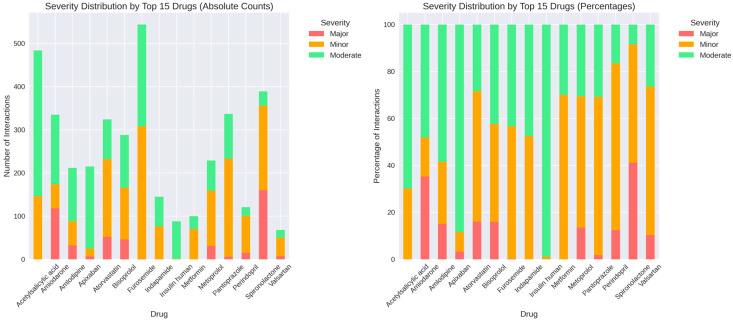
Severity distribution by top 15 medications. (**Left panel**): absolute counts of major, minor, and moderate interactions for each drug. (**Right panel**): percentage distribution showing relative se-verity composition, with notable variation across medications (e.g., pantoprazole predominantly minor interactions, while perindopril and spironolactone show higher proportions of major interactions).

The percentage distributions reflected the same ranking structure, with most medications showing moderate interactions as the largest proportion, except for several drugs (i.e., pantoprazole, perindopril and spironolactone), where minor interactions represented the majority. Major interactions accounted for a relatively small proportion across all medications, with the highest relative contributions observed for amiodarone, perindopril and spironolactone (Figure 7). 

**Figure 8:**
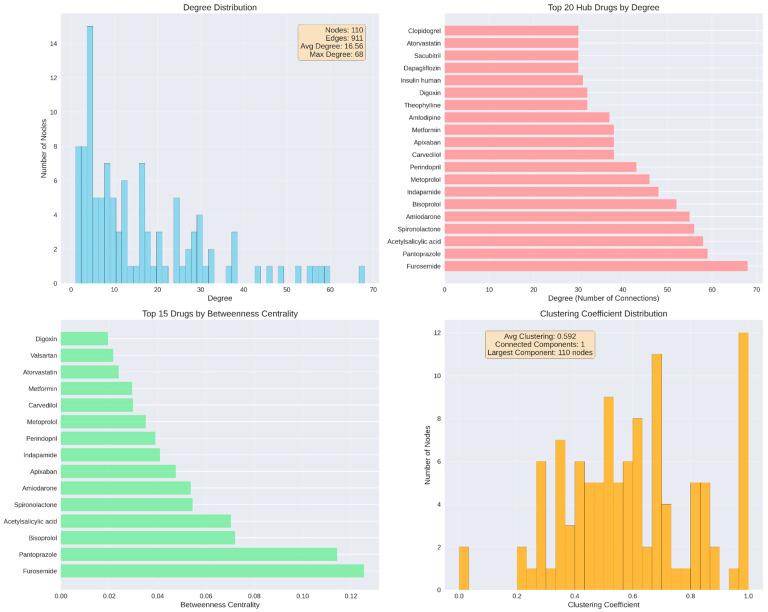
Network structure and hub drug identification. (**Top left**): degree distribution across 110 nodes and 911 edges. (**Top right**): top 20 hub drugs by degree, led by furosemide. (**Bottom left**): top 15 drugs by betweenness centrality, indicating bridging roles in the network. (**Bottom right**): clustering coefficient distribution, showing network cohesiveness with all nodes in a single connected component.

Network analysis identified 110 distinct medications connected through 911 interaction edges. The overall distribution of node degrees showed substantial variability across the network, with degree values ranging from 1 to a maximum of 68 and an average degree of 16.58. Lower-degree nodes were the most frequent, while higher-degree nodes occurred less often, with only a small number of medications reaching degrees above 40. Examination of the top 20 medications ranked by degree revealed furosemide as the most connected drug, followed by pantoprazole, acetylsalicylic acid, spironolactone, amiodarone, bisoprolol, indapamide, perindopril, carvedilol, metoprolol, amlodipine, apixaban, metformin, insulin human, theophylline, digoxin, dapagliflozin, sacubitril, and clopidogrel, with degrees progressively decreasing across this group. Betweenness centrality values varied across medications, with top-ranking drugs showing values spanning from 0.12 to 0.02. The distribution of clustering coefficients ranged between 0.2 and 0.8, with an average of 0.592. All nodes belonged to a single connected component encompassing all 110 medications (Figure 8).

Beyond degree and betweenness centrality, closeness centrality values ranged from 0.45 to 0.72, indicating varying diffusion potential within the interaction network. Eigenvector centrality values ranged from near-zero to 0.23, reflecting differential influence based on connections to other well-connected nodes. The overall network density was 0.152, indicating a moderately sparse structure in which only 15% of all possible drug–drug connections were present.

**Figure 9:**
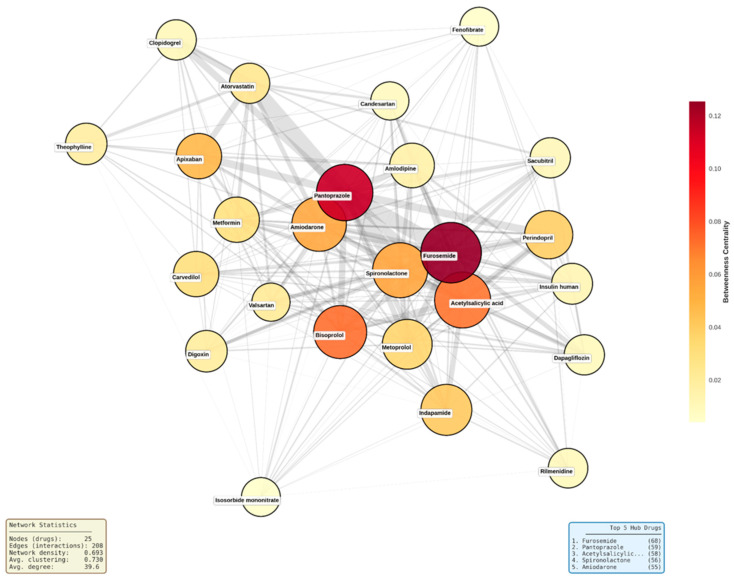
Network topology map of top 25 hub medications. Node size represents degree centrality (number of connections), node color represents betweenness centrality (yellow to red gradient indicates low to high bridging role), and edge width represents interaction count between drug pairs. Network density = 0.693, average clustering coefficient = 0.730. ‘Acetylsalicilic...’ refers to acetylsalicylic acid (text truncated in original figure).

Topological visualization of the 25 most connected drugs revealed an exceptionally dense interaction network with 208 edges connecting the hub medications, corresponding to a network density of 0.693 and an average clustering coefficient of 0.730 (Figure 9). This indicates that 69% of all possible drug pairs within this subset exhibited documented interactions, and when a drug interacted with two other medications, those medications were themselves highly likely to interact. Furosemide occupied the most central position, displaying both the highest degree centrality (68 connections) and highest betweenness centrality (0.124), followed closely by pantoprazole (degree 59, betweenness 0.120). Additional high-betweenness medications included acetylsalicylic acid (0.099), spironolactone (0.091), and bisoprolol (0.080), positioning these drugs as critical structural bridges within the polypharmacy network.

The network structure revealed a dense central core comprising approximately 15 drugs with near-complete interconnectivity, including furosemide, bisoprolol, metoprolol, spironolactone, and perindopril. Moderately connected drugs such as atorvastatin, clopidogrel, and valsartan occupied intermediate positions, while lower-degree medications like insulin human appeared at network peripheries. Edge thickness patterns indicated that the strongest interaction densities occurred between diuretics, between gastroprotective agents and antiplatelet drugs (pantoprazole with acetylsalicylic acid and clopidogrel), and among core cardiovascular medications, reflecting guideline-recommended combination therapy patterns that inherently generate multiple simultaneous interactions.

A positive association was observed between the total number of interactions and the number of major interactions (r = 0.763). This correlation coefficient was calculated in Python as part of exploratory descriptive analysis, without the use of hypothesis-testing or significance evaluation. Patients with higher overall interaction counts tended to display higher major interaction counts, as illustrated by the upward trend in the scatterplot.

**Figure 10:**
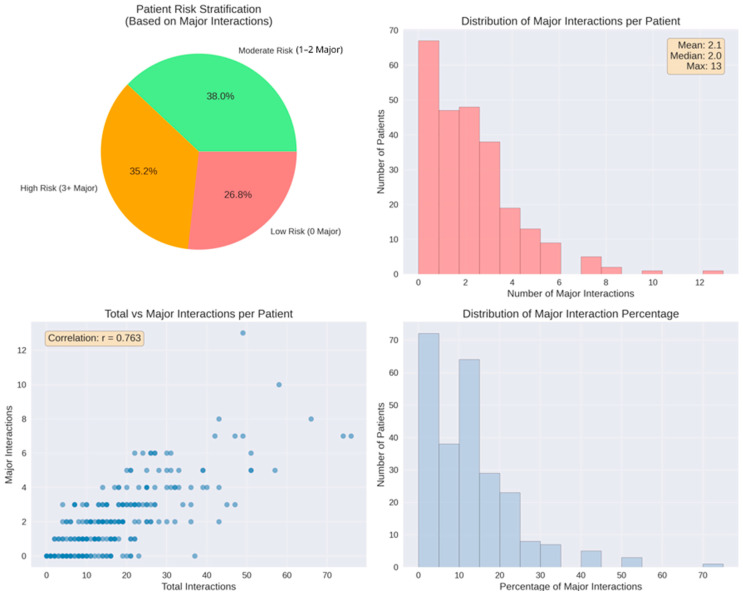
Patient-level major interaction analysis. Top left: risk stratification, 38.0% moderate risk (1–2 major), and 35.2% high risk (≥3 major). Top right: distribution with mean 2.1 major interactions per patient (range 0–13). Bottom left: positive correlation between total and major interactions (r = 0.763). Bottom right: distribution of major interaction percentage relative to total burden.

The proportion of major interactions relative to each patient’s total interaction burden demonstrated a marked right-skewed distribution. Most patients had low percentages of major interactions, with values predominantly falling below 20%. A smaller subset reached higher proportions, extending up to approximately 70% (Figure 10).

To assess the clinical relevance of the reported DDI burden (Figure 10), patient characteristics were stratified according to risk category (Table 3). The high-risk group (≥3 major interactions) represented a distinct clinical phenotype characterized by advanced age (70.1 ± 12.0 years) and intensive polypharmacy (mean 9.8 medications). Clinically, this group was dominated by patients with chronic heart failure (37.5%), reflecting the high interaction potential of guideline-directed heart failure regimens (e.g., mineralocorticoid receptor antagonists combined with renin-angiotensin system inhibitors).

In contrast, the low-risk group displayed a different diagnostic profile, with unstable angina being the most frequent diagnosis (32.8%). These patients, despite being on acute cardiovascular protocols, typically received standardized antiplatelet-statin regimens that, while interactive, accumulated fewer major severity flags compared to the complex diuretic-based schemes seen in the high-risk cohort.

## 4. Discussion

DDIs represent a critical challenge in contemporary cardiovascular medicine, particularly among elderly patients experiencing multimorbidity and polypharmacy. Recent epidemiological evidence demonstrates that potential DDIs are remarkably prevalent in cardiovascular populations, with studies reporting that 77.5% to 95% of hospitalized elderly patients with cardiovascular disease experience at least one severe potential interaction [12,29]. In a recent prospective analysis of cardiology ward admissions, 88% of all identified DDIs involved cardiovascular medications, underscoring the inherent interaction vulnerability embedded within routine cardiac pharmacotherapy [30]. These findings are further corroborated by contemporary research in atrial fibrillation cohorts, where 69% of patients exhibited at least one clinically relevant potential DDI at discharge [29]. Together, these data indicate that DDI exposure is not an exceptional event but a routine consequence of contemporary cardiovascular care. The relationship between medication burden and interaction risk follows predictable mathematical principles. As the number of concurrently prescribed agents increases, the probability of DDI formation rises exponentially according to combinatorial scaling [31]. 

Studies consistently demonstrate that polypharmacy, defined variably as five or more concurrent medications, affects 74.8% to 97.2% of elderly cardiovascular patients [29,32]. This high medication burden reflects a therapeutic paradox, as evidence-based cardiovascular management requires multiple agents to optimize outcomes while simultaneously increasing exposure to DDIs and adverse drug reactions (ADR) [14]. Clinically, DDIs contribute to acute medical admissions among elderly patients [33] and represent a major source of preventable medication errors, with global costs exceeding $42 billion annually [34]. Within cardiovascular pharmacotherapy, antiplatelet agents, anticoagulants, diuretics, and renin–angiotensin system inhibitors are particularly prone to interaction-related harm due to narrow therapeutic windows and significant risk of bleeding, electrolyte disturbances, and acute kidney injury [35,36].

Outcome-based evidence from hospitalized cohorts further supports the clinical relevance of DDI burden. In a large retrospective analysis of hospitalized patients, Gallelli et al. reported that ~22% of documented ADRs were attributable to DDIs using a probability-based causality framework and that higher medication counts were associated with ADR occurrence. While our study focuses on potential DDIs and does not assess ADRs or therapeutic drug monitoring, these findings provide external clinical context for why mapping DDI accumulation and network hubs is relevant for risk prevention [37].

Digital drug interaction screening tools provide rapid and systematic assessment of potential DDIs at the point of care [34]. Platforms such as Drugs.com, Lexicomp, and Medscape integrate extensive pharmacological databases and offer standardized severity classifications and management recommendations [10,18]. Drugs.com, used in the present study, demonstrates high sensitivity (>90%) for clinically significant DDIs and provides detailed mechanistic and management information [38]. Although concordance varies between databases, these tools are widely recognized as essential decision-support systems for managing polypharmacy-related risk [16,39]. However, the availability of screening tools alone does not address the cumulative and structural nature of the DDI burden in cardiovascular polypharmacy, which provided the rationale for the present investigation.

The demographic profile of the analyzed cohort framed the clinical context in which DDIs emerged. Most patients were older adults, with a mean age above 68 years and a substantial clustering in the seventh and eighth decades of life. This age structure is inherently associated with multimorbidity, prolonged disease duration, and cumulative pharmacological exposure, all of which favor regimen expansion. The relatively balanced sex distribution indicates that DDI patterns were driven primarily by age-related complexity rather than sex-specific prescribing behaviors. As such, the baseline characteristics already indicated a population inherently predisposed to polypharmacy and interaction vulnerability.

The findings of the present study align with and extend existing evidence on DDIs in cardiovascular populations. Polypharmacy prevalence in this cohort, with patients receiving nearly eight medications on average, closely mirrors contemporary epidemiological data [40,41]. This concordance supports the representativeness of the cohort and indicates that the observed interaction burden reflects routine cardiovascular inpatient care rather than an exceptional high-risk subgroup. Notably, medication burden accumulation followed distinct trajectories between sexes. Male patients demonstrated a clear age-dependent increase in polypharmacy, with medication counts nearly doubling between the youngest and oldest age groups. In contrast, female patients maintained consistently high medication counts across all age categories, resulting in a significant age × sex interaction (*p* = 0.002). This pattern suggests that factors driving polypharmacy accumulation may differ between sexes, with female patients potentially experiencing earlier multimorbidity onset or more aggressive pharmacological management of cardiovascular risk factors independent of age.

Medication distribution further reinforced this interpretation. Patients received an average of nearly eight medications at admission. Most patients fell into medium- to high-medication-burden categories, while only a minority received fewer than five drugs. Because the number of pharmacological agents directly determines the number of possible interaction pairs, this baseline medication burden critically shapes downstream DDI patterns. The scarcity of low-medication patients underscores that interaction risk is embedded in routine cardiovascular management.

A key quantitative finding was the strong correlation between medication count and total interactions (r = 0.929). This linear association reflects predictable combinatorial scaling, whereby each additional drug substantially increases the number of potential interaction pairs. Clinically, this demonstrates that interaction accumulation is an inherent consequence of therapeutic expansion rather than isolated prescribing error. This relationship is consistent with the mathematical principle *n*(*n* − 1)/2 [31] and has been validated across diverse healthcare systems [42]. Our findings empirically validate this theoretical framework within an acute cardiovascular care environment.

Pantoprazole, furosemide, spironolactone, acetylsalicylic acid, and atorvastatin accounted for the highest number of interactions. This pattern reflects medication prevalence rather than intrinsic interaction severity. Pantoprazole is frequently co-prescribed for gastroprotection, while furosemide and spironolactone are cornerstone therapies in heart failure, ensuring their presence across multiple regimens. Accordingly, high interaction counts primarily indicate structural centrality within standard cardiovascular therapy rather than disproportionate pharmacological hazard.

Pantoprazole’s dominant interaction footprint aligns with widespread proton pump inhibitor use in cardiovascular populations, often exceeding 60% of patients [43]. Its interaction profile is clinically relevant, particularly through CYP2C19-mediated effects on antiplatelet therapy, most notably clopidogrel [44,45]. While guideline discussions continue regarding relative safety among PPIs [46], the recurrence of pantoprazole-based combinations highlights deprescribing opportunities, given persistent overuse without clear indications [47]. Thus, pantoprazole represents a high-impact, modifiable hub within the interaction network rather than an inherently high-risk drug.

Drug pair analysis demonstrated high diversity, with most of the 911 unique combinations occurring infrequently. However, a limited subset of recurrent pairs consistently shaped the interaction core. These combinations reflect routine prescribing sequences, such as atorvastatin + pantoprazole, furosemide + pantoprazole, or pantoprazole + spironolactone regimens. This pattern indicates that DDI burden arises from standardized therapeutic pathways rather than random drug selection.

Furosemide emerged as a major interaction contributor, consistent with its central role in heart failure management. Unlike pantoprazole, its interactions are predominantly pharmacodynamic, involving electrolyte imbalance, renal function, and ototoxicity potentiation. Accordingly, most furosemide-related DDIs are manageable through monitoring rather than avoidance, in line with guideline recommendations [48,49]. This profile illustrates how high interaction frequency does not necessarily equate to high clinical severity.

Spironolactone contributed disproportionately to major-severity interactions. This finding reflects its narrow therapeutic window and potassium-retaining effects, particularly when combined with renin–angiotensin system inhibitors or nonsteroidal anti-inflammatory drugs (NSAIDs) [50,51]. Hyperkalemia remains a leading serious adverse event in heart failure management [52]. Our observation that 73.2% of patients experienced at least one major interaction, with spironolactone frequently involved, parallels registry data identifying mineralocorticoid receptor antagonists as strong predictors of high-severity DDI burden [53].

Atorvastatin’s prominent interaction profile reflects its widespread use. Although many statin-related DDIs are pharmacokinetic, clinically significant toxicity remains relatively uncommon, occurring in fewer than 5% of patients [54]. Statin-related DDIs present a complex risk–benefit calculus due to metabolism primarily via the CYP3A4 pathway, creating potential interactions with numerous commonly prescribed cardiovascular medications, including calcium channel blockers, amiodarone, and certain antifungal agents [55,56]. The frequent atorvastatin–pantoprazole combination illustrates the disconnect between theoretical interaction risk and clinical expression [57], highlighting limitations of pairwise DDI interpretation.

Acetylsalicylic acid appeared frequently due to its ubiquitous role in cardiovascular prevention. Its primary interaction risk relates to bleeding potentiation rather than pharmacokinetic interference [58]. While bleeding risk is clinically relevant, it is generally managed through dose optimization and individualized risk–benefit assessment rather than avoidance [59].

Sex-specific prescribing patterns were observed for oral anticoagulants, with rivaroxaban demonstrating female predominance (male/female ratio 0.61) while apixaban exhibited the opposite pattern (ratio 1.72). This divergence may reflect prescriber preferences influenced by patient characteristics such as renal function, body weight, or bleeding risk profiles, which differ between sexes and influence anticoagulant selection. These patterns warrant further investigation to determine whether sex-specific DDI profiles emerge from differential anticoagulant prescribing.

Severity analysis further refined clinical interpretation. Minor and moderate interactions each accounted for approximately 44% of events, while major interactions represented 12.1%. Importantly, 73.2% of patients experienced at least one major interaction, and over one-third met criteria for high-risk exposure (≥3 major interactions). These findings indicate that clinically significant interaction risk is concentrated in a substantial subgroup rather than being uniformly distributed and aligns with recent multicenter data [60]. However, severity distributions vary substantially across clinical contexts, with ambulatory settings typically demonstrating lower proportions of major interactions compared to acute care environments where medication intensity is higher [61]. This concentration of risk underscores the importance of targeted rather than generalized mitigation strategies. Notably, the youngest cohort (<50 years) exhibited the highest relative proportion of major interactions (14.4%), exceeding that of even the oldest age group (13.0%). This counterintuitive finding may reflect the higher-intensity pharmacotherapy required in younger patients presenting with acute cardiovascular events, where aggressive dual antiplatelet therapy, anticoagulation, and heart failure regimens are initiated simultaneously. In contrast, elderly patients may be managed with greater pharmacological caution due to concerns about frailty and ADRs, resulting in less intensive but more numerous minor and moderate interactions.

Amiodarone demonstrated a concentrated high-severity interaction profile. Its long half-life, extensive tissue accumulation, and multi-pathway cytochrome P450 inhibition generate persistent interaction risk [62]. Amiodarone interactions include QT prolongation potentiation with numerous cardiac and non-cardiac medications, warfarin potentiation requiring dose reduction in virtually all patients, and increased digoxin levels through P-glycoprotein inhibition [63]. These properties explain its strong association with severe DDIs despite moderate use frequency [64].

Perindopril presents a well-established interaction profile of angiotensin-converting enzyme inhibitors with potassium-sparing agents, NSAIDs, and direct renin inhibitors. Perindopril’s major interaction contribution reflects known risks associated with potassium-altering combinations. The tension between mortality-reducing therapy and interaction-related toxicity represents a core challenge in guideline-directed care [65,66].

Network analysis elucidated the structural basis of these findings. The interaction network formed a single connected component, indicating that no medication was isolated from potential DDIs. High-degree and high-betweenness hubs functioned as structural bridges linking therapeutic clusters, demonstrating that interaction risk is systemically embedded within guideline-directed cardiovascular therapy rather than driven by isolated problematic agents.

Beyond global metrics, the markedly higher density and clustering observed in the core cardiovascular subnetwork compared with the full network explain the mathematical inevitability of DDI accumulation as regimens expand. Each additional drug enters an already saturated interaction field, amplifying cumulative burden rather than forming isolated pairs.

The network structure revealed three organizationally distinct features with direct clinical implications. First, the dense central core comprising furosemide, bisoprolol, metoprolol, spironolactone, perindopril, and related cardiovascular agents exhibited near-complete interconnectivity, indicating that standard heart failure and hypertension regimens unavoidably generate multiple concurrent DDIs regardless of prescriber intent. Second, the elevated clustering coefficient (0.730) demonstrates that when a medication interacts with two others, those medications themselves are highly likely to interact, creating interaction cascades rather than isolated pairwise events. This clustering phenomenon means that therapeutic expansion triggers multiplicative rather than additive DDI burden, consistent with the exponential scaling observed between medication count and interaction frequency. Third, the absence of a hub-and-spoke topology wherein a single problematic medication would serve as the central connector reveals that cardiovascular DDI risk is systemically embedded within guideline-directed combination therapy rather than attributable to specific avoidable agents. 

The present study applies comparable graph-theoretic algorithms to a hospitalized cardiovascular cohort, identifying a markedly disproportionate interaction density among the core highly connected medications compared to the comparative sparsity of the overall network. This architectural distinction between core therapeutic agents and the broader medication landscape aligns with recent clinical observations emphasizing the inherent complexity of guideline-directed cardiovascular polypharmacy. The European Society of Cardiology Heart Failure Association has specifically highlighted the need for dedicated polypharmacy management strategies in heart failure populations, noting that DDIs are common when patients are prescribed multiple treatments and advocating for heart failure-specific tools to address therapeutic complexity [15].

In contrast to prior studies relying predominantly on interaction frequencies alone, the present network-based approach demonstrates that interaction risk is not randomly distributed but follows reproducible topological patterns driven by core cardiovascular therapies. The network density of 0.152 observed in our study indicates moderate interconnectedness, while the clustering coefficient of 0.592 reveals substantial grouping of medications into clinically coherent clusters such as antihypertensive–diuretic–PPI combinations. This structural organization suggests that interaction risk emerges from consistent therapeutic patterns inherent to cardiovascular management rather than random drug mixing [67,68].

Patient-level risk stratification demonstrated substantial heterogeneity. While 26.8% had no major interactions, more than a third exhibited high-risk profiles. The wide variation in the proportion of major interactions per patient, ranging up to ~70%, illustrated that clinical danger is shaped not only by absolute interaction numbers but also by the relative concentration of high-severity events within each regimen.

Risk mitigation strategies must prioritize systematic monitoring protocols, rational therapeutic intensity assessment, and structured deprescribing rather than simple avoidance of individual high-risk medications, as the latter approach is incompatible with evidence-based cardiovascular management.

Stratification of patient characteristics by DDI risk category (Table 2) revealed distinct clinical phenotypes. High-risk patients (≥3 major interactions) were characterized by advanced age (70.1 ± 12.0 years), male predominance (60.2%), and a high prevalence of chronic heart failure (37.5%). This profile reflects the inherent interaction complexity of guideline-directed heart failure therapy, which mandates concurrent use of mineralocorticoid receptor antagonists, renin-angiotensin system inhibitors, and diuretics, a combination systematically generating major DDIs. Conversely, low-risk patients were younger, more frequently diagnosed with unstable angina (32.8%), and treated with standardized antiplatelet-statin regimens that, while interactive, accumulate fewer high-severity flags. These findings support the development of diagnosis-specific DDI surveillance protocols, with intensified monitoring prioritized for heart failure patients.

Deprescribing proton pump inhibitors represents a major opportunity, given their central position within the interaction network and their frequent inappropriate use. Between 25 and 70% of PPI prescriptions lack a valid indication, particularly in cardiovascular settings [69]. In the pantoprazole-dominant interaction profile observed in this cohort, structured reassessment of ongoing PPI indication, particularly in the absence of Barrett esophagus, severe esophagitis, or prior gastrointestinal bleeding, could substantially reduce overall DDI burden without compromising gastroprotection.

Targeted potassium monitoring is essential in spironolactone-containing regimens due to their disproportionate contribution to major-severity DDIs. Real-world studies show that hyperkalemia risk associated with spironolactone combined with angiotensin-converting enzyme inhibitors or angiotensin receptor blockers exceeds rates observed in clinical trials, underscoring the need for intensified surveillance in routine practice [50]. Systematic potassium monitoring has been shown to reduce hyperkalemia-related events by approximately 50%, with recommended monitoring intervals of every 4–12 weeks for potassium ≤4.5 mEq/L, every 2–4 weeks for levels between 4.6 and 5.0 mEq/L, and more frequent assessment when levels exceed 5.0 mEq/L after therapy initiation [70]. Dose minimization remains critical, as 25 mg daily is associated with significantly lower hyperkalemia risk than 50 mg daily [71]. In high-risk patients from the present cohort (≥3 major DDIs involving spironolactone), this approach should be reinforced through laboratory surveillance and structured patient education regarding dietary potassium and symptom recognition.

Amiodarone requires dedicated safety protocols due to its complex pharmacokinetic profile. Recommended strategies include baseline and periodic monitoring of thyroid, hepatic, pulmonary, and ophthalmologic function, careful INR monitoring in patients receiving warfarin, and empiric digoxin dose reduction by approximately 50% upon amiodarone initiation [72,73]. Maintenance at the lowest effective dose and routine electrocardiographic monitoring for QT prolongation remain essential. Heightened vigilance is particularly warranted when amiodarone is co-administered with other electrophysiologically active agents, especially in evolving therapeutic contexts such as SGLT2 inhibitors and GLP-1 receptor agonists, where electrolyte balance plays a critical role [62]. In the present cohort, structured multidisciplinary collaboration between cardiologists and clinical pharmacists, coupled with systematic DDI screening at each therapeutic modification, represents optimal risk mitigation.

Integration of computerized clinical decision support systems (CDSS) combined with active pharmacist review can reduce medication errors by approximately 50%, provided alert systems are optimized to limit alert fatigue while preserving sensitivity for clinically significant interactions [74,75]. Strategies such as severity-based alert stratification and context-dependent suppression of low-relevance notifications have achieved reductions of 55% in alert volume and 45% in pharmacist workload without compromising safety [76]. In highly polymedicated cardiovascular populations, effective systems should prioritize high-severity alerts linked to mandatory pharmacist intervention while delivering lower-severity guidance in a non-disruptive manner.

Finally, leveraging network analysis metrics enables targeted, preventive interventions focused on pharmacological hubs. Medications with high degree centrality (furosemide, pantoprazole, spironolactone), high betweenness centrality (furosemide, bisoprolol), and elevated eigenvector centrality represent priority targets for proactive monitoring, rational therapeutic substitution, and deprescribing strategies. Network-based drug interaction modeling has demonstrated the ability to identify high-risk combinations and anticipate interaction propagation within complex therapeutic systems [77]. 

Beyond internal validity, the extent to which these findings reflect cardiovascular prescribing patterns across different healthcare systems is an important consideration. Although this study was conducted in a single tertiary hospital in Romania, several features support the generalizability of the findings to cardiovascular populations in other countries. 

First, the mean medication burden of 7.7 drugs per patient is consistent with international data from hospitalized cardiovascular cohorts. Studies from Western Europe and North America commonly report average medication counts between 7 and 9 drugs per patient, with even higher values frequently observed among older patients with heart failure [11,12,31]. Similarly, data from Asia indicate comparable prescribing intensity, as patients with atrial fibrillation in China were reported to receive a median of 7 medications (interquartile range 4–10) [29].

Second, the near-universal prevalence of potential DDIs (98.4%) observed in our cohort closely aligns with international estimates. Reports from European cardiovascular pharmacotherapy groups indicate that up to 90% of elderly multimorbid patients are exposed to at least one potential DDI, while approximately one-third experience high-risk interaction profiles [14]. Accordingly, the proportion of patients with ≥3 major interactions (35.2%) in the present study falls well within ranges reported across diverse healthcare systems, supporting external validity [12,30].

Third, the observed severity distribution (approximately 44% minor, 44% moderate, and 12% major interactions) is consistent with findings from international DDI surveillance studies using commonly applied interaction databases such as Drugs.com, Lexicomp, and Micromedex. Across cardiovascular inpatient populations, major interactions typically account for 8–15% of detected DDIs, indicating that the risk profile identified here may reflect global prescribing patterns rather than local anomalies [30].

Fourth, the medications identified as central hubs within the interaction network, including proton pump inhibitors, loop diuretics, mineralocorticoid receptor antagonists, renin–angiotensin system inhibitors, statins, and antiarrhythmic agents, represent core components of guideline-directed cardiovascular therapy worldwide. These agents are consistently recommended across major international cardiovascular guidelines, and their prominence within the interaction network therefore reflects universal therapeutic strategies rather than country-specific prescribing practices [14,15]. The numerical patterns, interaction severity profiles, and structural drivers of DDI burden observed in this study are likely generalizable to diverse cardiovascular care settings, despite the single-center design.

### Strengths and Limitations

The main strength of this study lies in the integration of comprehensive DDI profiling with graph-theoretic network analysis in a real-world cohort of hospitalized cardiovascular patients. This approach enabled identification of not only interaction frequency and severity but also the structural role of hub medications and clustered therapeutic patterns that shape cumulative DDI burden. The use of patient-level data from routine clinical practice enhances the clinical relevance of the findings and supports their applicability to contemporary cardiovascular care.

Several limitations should be acknowledged beyond the size of the patient cohort. The analysis identifies potential DDIs and therefore cannot establish clinical causality or confirm adverse outcomes. As this was a retrospective observational study based on admission medication lists, no prospective monitoring or adjudication of DDI-related clinical events was performed. During the observed hospitalization period, no systematically documented serious adverse outcomes could be directly attributed to specific DDI pairs. Importantly, the study did not include systematic assessment of clinical outcomes or therapeutic drug monitoring. Therefore, the clinical expression and real-world consequences of identified DDIs could not be evaluated. Reliance on a single interaction database may introduce classification variability despite its high sensitivity. The absence of pharmacogenomic data and precise temporal information on drug exposure limits individualized interpretation of interaction severity. The single-center design may restrict generalizability, although the cohort characteristics are consistent with those reported in other cardiovascular inpatient populations. The absence of systematically recorded lifestyle variables and detailed comorbidity indices limited the evaluation of their potential association with DDI burden.

## 5. Conclusions

In this real-world cohort of hospitalized cardiovascular patients, polypharmacy was highly prevalent (mean 7.7 medications per patient), and exposure to potential DDIs was nearly universal, with 98.4% of patients presenting at least one interaction and 35.2% exhibiting high-risk profiles (≥3 major DDIs). The strong association between medication count and interaction burden (r = 0.929) indicates that DDI accumulation is an inherent feature of contemporary cardiovascular pharmacotherapy. 

Network-based analysis demonstrated that a limited set of hub medications disproportionately drive both overall interaction burden and major-severity risk. These findings support a shift from isolated pairwise DDI screening toward network-informed mitigation strategies focused on hub drugs, including targeted monitoring, rational deprescribing, and optimized clinical decision support. Future research should link potential DDIs to clinical outcomes and incorporate predictive models while also integrating lifestyle factors and broader patient-specific modifiers in larger, multicenter cohorts to improve individualized DDI risk assessment.

## Figures and Tables

**Table 1:** Summary of network analysis metrics, implementation, and interpretation.

Metric	NetworkX Implementation	Description	Relevance
Degree centrality	nx.degree()	Number of direct interactions per drug	Identifies highly interactive hub medications
Betweenness centrality	nx.betweenness_centrality()	Frequency of a drug acting as a bridge	Detects drugs propagating interaction risk
Closeness centrality	nx.closeness_centrality()	Inverse mean distance to all nodes	Reflects diffusion potential of DDIs
Eigenvector centrality	nx.eigenvector_centrality()	Influence within highly connected subnetworks	Identifies structurally influential drugs
Network density	nx.density()	Proportion of realized edges	Quantifies overall interaction saturation
Clustering coefficient	nx.clustering() nx.average_clustering()	Local and global interconnectivity	Indicates interaction cascade potential
Hub subnetwork visualization	nx.kamada_kawai_layout()	Subgraph of top 25 drugs by degree centrality	Highlights densely interconnected core driving cumulative DDI burden

DDIs, drug–drug interactions.

**Table 2:** The demographic and clinical characteristics.

Characteristic	Total (*n* = 250)	Male (*n* = 139)	Female (*n* = 111)
Age, years			
Mean ± SD	68.27 ± 12.80	66.03 ± 13.13	71.08 ± 11.85
Median (range)	70 (24–94)	67 (24–91)	72 (39–94)
Residence, *n* (%) *			
Urban	98 (39.2%)	57 (41.0%)	41 (36.9%)
Rural	152 (60.8%)	81 (58.3%)	71 (64.0%)
Number of medications			
Mean ± SD	7.70 ± 2.73	7.86 ± 2.85	7.50 ± 2.58
Median (range)	8 (1–16)	8 (1–16)	7 (1–14)
Primary cardiovascular diagnosis, *n* (%) *			
Heart failure	57 (22.8%)	33 (23.7%)	24 (21.6%)
Unstable angina	52 (20.8%)	30 (21.6%)	22 (19.8%)
Atrial fibrillation	32 (12.8%)	20 (14.4%)	12 (10.8%)
Acute myocardial infarction	30 (12.0%)	19 (13.7%)	11 (9.9%)
Pulmonary embolism	27 (10.8%)	10 (7.2%)	17 (15.3%)
Hypertension	20 (8.0%)	8 (5.8%)	12 (10.8%)
Acute pulmonary edema	6 (2.4%)	4 (2.9%)	2 (1.8%)
Atrioventricular block	5 (2.0%)	3 (2.2%)	2 (1.8%)
Ventricular tachycardia	4 (1.6%)	4 (2.9%)	0 (0.0%)
Venous thrombosis	4 (1.6%)	3 (2.2%)	1 (0.9%)
Acute coronary syndrome	3 (1.2%)	1 (0.7%)	2 (1.8%)
Sick sinus syndrome	3 (1.2%)	0 (0.0%)	3 (2.7%)
Ischemic cardiopathy	2 (0.8%)	1 (0.7%)	1 (0.9%)
Aortic dissection	1 (0.4%)	1 (0.7%)	0 (0.0%)
Atrial flutter	1 (0.4%)	1 (0.7%)	0 (0.0%)
Infective endocarditis	1 (0.4%)	0 (0.0%)	1 (0.9%)
Supraventricular tachycardia	1 (0.4%)	1 (0.7%)	0 (0.0%)
Cardiac tamponade	1 (0.4%)	0 (0.0%)	1 (0.9%)

SD, standard deviation; *, percentages may not sum to 100% due to rounding.

**Table 3:** Comparison of patient demographics, comorbidities, and medication burden across low, moderate, and high drug–drug interaction (DDI) risk categories.

Variable	Low Risk	Moderate Risk	High Risk
*n* (%)	67 (26.8%)	95 (38.0%)	88 (35.2%)
Age, years (Mean ± SD)	65.6 ± 14.3	68.4 ± 12.2	70.1 ± 12.0
Male, *n* (%)	34 (50.7%)	52 (54.7%)	53 (60.2%)
Female, *n* (%)	33 (49.3%)	43 (45.3%)	35 (39.8%)
Chronic Heart Failure, *n* (%)	5 (7.5%)	19 (20.0%)	33 (37.5%)
Hypertension, *n* (%)	11 (16.4%)	7 (7.4%)	2 (2.3%)
Atrial Fibrillation, *n* (%)	5 (7.5%)	17 (17.9%)	10 (11.4%)
Unstable Angina, *n* (%)	22 (32.8%)	20 (21.1%)	10 (11.4%)
Acute Myocardial Infarction, *n* (%)	4 (6.0%)	14 (14.7%)	12 (13.6%)
Number of Medications (Mean ± SD)	5.6 ± 2.2	7.2 ± 1.8	9.8 ± 2.5
Major Interactions (Mean ± SD)	0.0 ± 0.0	1.5 ± 0.5	4.4 ± 1.8

## Data Availability

The original contributions presented in this study are included in the article. Further inquiries can be directed to the corresponding authors.

## Abbreviations

The following abbreviations are used in this manuscript:

DDI	Drug–drug interaction
PPI	Proton pump inhibitor
NSAID	Nonsteroidal anti-inflammatory drug
ACE	Angiotensin-converting enzyme
CDSS	Clinical decision support system
CVD	Cardiovascular disease
ADR	Adverse drug reaction
SGLT2	Sodium-glucose cotransporter-2
GLP-1	Glucagon-like peptide-1
